# Social vulnerability and spatial patterns of COVID-19 mortality: Global implications for respiratory health equity

**DOI:** 10.1371/journal.pone.0352270

**Published:** 2026-07-01

**Authors:** Gregory D. Kearney, Ogugua Ndili Obi, Veeranna Maddipati, Gregory Levitin, Guangxiao Hu

**Affiliations:** 1 Department of Public Health, Brody School of Medicine, East Carolina University, Greenville, North Carolina, United States of America; 2 Division of Pulmonary, Critical Care and Sleep Medicine, Brody School of Medicine, East Carolina University, Greenville, North Carolina, United States of America; 3 Department of Otolaryngology, Icahn School of Medicine at Mount Sinai, New York, New York, United States of America; 4 Department of Earth, Environment, and Planning, Thomas Harriot College, East Carolina University, Greenville, North Carolina, United States of America; Syracuse University, UNITED STATES OF AMERICA

## Abstract

**Background:**

The pandemic highlighted geographic inequities in COVID-19 mortality worldwide, reflecting structural and social vulnerabilities that exacerbated respiratory disease outcomes. The primary aim of this study was to examine the spatial relationship between COVID-19 mortality and communities characterized by greater social disadvantage in North Carolina. This application highlights the value of spatial analytic approaches when considering inequities in respiratory mortality.

**Methods:**

This study analyzed 25,051 COVID-19 deaths occurring in North Carolina from March 2020 through April 2022 using state vital statistics data and the Centers for Disease Control and Prevention, Social Vulnerability Index data. SaTScan™ was used to identify mortality hot spots, and Moran’s I in ArcGIS Pro was used to assess spatial autocorrelation at the ZIP-Code Tabulation Area level. The Mann–Whitney U test was used to assess differences in social vulnerability index scores in mortality hot spots areas and non-hot spots areas.

**Results:**

Local bivariate analysis identified moderate spatial clustering characterized by high–high (mortality–vulnerability) hot spots and spatial outliers. In non-parametric testing, COVID-19 mortality hot spot areas had significantly higher, but modestly elevated, median overall social vulnerability index scores compared with non–hot spot areas (0.58 vs. 0.45; *p* < 0.001).

**Conclusions:**

Social vulnerability partially explains the observed spatial heterogeneity in COVID-19 mortality (2020–2022), with statistically significant spatial clustering consistently concentrated in areas of higher social vulnerability. This study highlights how pre-existing inequities in healthcare access and social conditions intensified mortality disparities during the pandemic.

## Introduction

The Coronavirus Disease 2019 (COVID-19) pandemic strained healthcare systems across the United States, exposing longstanding structural weaknesses in access, resource allocation, and population health outcomes [1,2]. These vulnerabilities disproportionately affected racial and ethnic minorities, socioeconomically populations, immigrants, and individuals living or working in high exposure environments, including congregate and occupational settings [3,4]. As the pandemic unfolded, early media coverage and public discourse emphasized as the primary risk factor for disease severity and mortality while comparatively less attention was given to the cumulative role of structural and social determinants of health [3–6]. This overemphasis on age diverted attention from broader social and economic conditions shaping population-level risk such as poverty, limited access to healthcare, delayed vaccine uptake [3–6].

North Carolina (N.C.) represents a salient case study of COVID-19 inequities, characterized by pronounced racial and ethnic disparities among underrepresented minorities, rooted in historical patterns of enslavement, segregation, as well as low socioeconomic status workforce concentrated in agriculture and other high-risk essential occupations [6–8]. Consistent with these structural conditions, early studies in North Carolina documented substantial racial, ethnic, and geographic disparities in COVID-19 burden. Surveillance data showed markedly higher SARS-CoV-2 test positivity among non-Hispanic Black (22.0%) and Hispanic (66.5%) residents compared with non-Hispanic White residents (11.5%), alongside higher population-adjusted case burdens (positive tests per 10,000: 42.7 and 106.0 vs 17.0, respectively) [8]. These disparities extended beyond infection risk to clinical severity, with rural and socioeconomically disadvantaged populations experiencing significantly worse hospitalization outcomes during successive pandemic waves [7]. Together, these inequities translated into disproportionate mortality, with COVID-19 death rates during January–September 2020 reported to be 1.6 times higher among Black residents and COVID-19 case rates 2.3 times higher among Hispanic residents compared with non-Hispanic Whites [9]. Together, these factors underscore the intersecting roles of race, geography, and socioeconomic status in shaping unequal pandemic outcomes.

As the pandemic unfolded across the U.S. public health agencies rapidly deployed interactive, public web-based dashboards, with visualization and mapping tools to support surveillance efforts and inform communities [10]. Although valuable for tracking and communicating overall COVID-19 trends, these tools had limitations. Analyses were typically conducted at coarse spatial resolutions, which frequently obscured small-area inequities [11]. Together, these limitations restricted mapping and visualization capabilities and hindered the ability to examine fine-scale geographic variation in COVID-19 risk and outcomes.

Given the strong influence of social determinants on COVID-19 outcomes, there remains a critical need for fine-scale, small-area spatial analyses using geographic information systems (GIS) to identify concentrated mortality patterns and their association with underlying social vulnerability. Such approaches provide essential insights to inform geographically targeted and equity-oriented public health preparedness and response strategies for future pandemic events [8,12].

This study had three objectives; (1) to evaluate spatial patterns and temporal trends of COVID-19 deaths across distinct timeframes during the pandemic; (2) identify statistically significant spatial clustering of hot spots of elevated mortality; and (3) assess spatial autocorrelation to determine whether deaths from COVID-19 were more likely to occur in areas considered more socially vulnerable. By applying spatial analytical methods to assess mortality patterns and trends, this study provides actionable evidence to inform equitable public health surveillance, targeted interventions, and data-driven pandemic preparedness and response.

## Methods

This was an ecological study using a spatial analysis framework to assess COVID-19 mortality patterns in N.C. from March 1, 2020, to April 30, 2022, a period encompassing all major pandemic waves. The analytic design and methods align with international standards in spatial epidemiology, allowing for replication across other national and subnational contexts. The study involved secondary analysis of de-identified, publicly available data and was determined exempt from human subjects review by East Carolina University, Institutional Review Board.

### Data collection

Mortality data were obtained from the N.C. Department of Health and Human Services (NCDHHS), State Center for Health Statistics (SCHS). Inclusion criteria comprised any N.C. resident whose underlying cause of death (UCD) was coded as COVID-19 (ICD-10 U07.1) during the study period. To capture community-level social and demographic vulnerability, we relied on the Centers for Disease Control and Prevention (CDC), Social Vulnerability Index (SVI) dataset [13,14]. The SVI is a place-based tool designed to quantify community vulnerability to external stressors using data derived from the U.S. Census Bureau, American Community Survey (ACS). Although originally developed as a tool for community disaster preparedness and response, the SVI tool has been widely adopted across diverse public health contexts [11,12,14,15]. Other comparable composite indices, such as the United Nations Development Program’s (UNDP) Multidimensional Vulnerability Index and the WHO Urban Health Index, further demonstrate the global applicability of such frameworks for identifying and monitoring socially vulnerable populations across countries [16].

**Fig 1:**
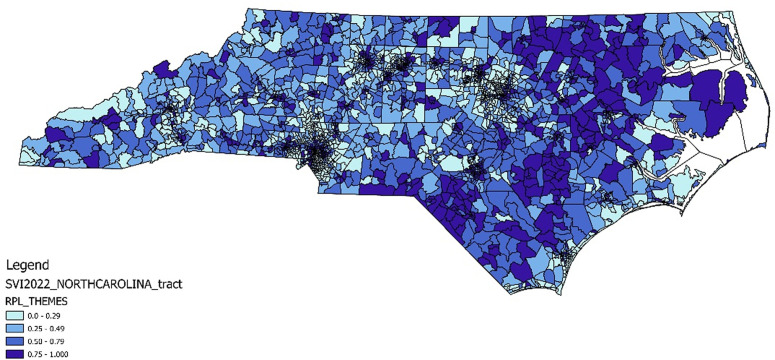
CDC, social vulnerability index map and values at the ZIP-code tabulation area level, North Carolina (2022). Note: This figure was created by the authors using ArcGIS Pro and is based exclusively on publicly available U.S. federal and state spatial data; no proprietary basemap or copyrighted imagery was used. Source: CDC/ATSDR, 2022 [13].

The SVI is based on 16 census-derived variables, grouped into four separate thematic domains: (i) socioeconomic status, (ii) household composition, (iii) racial/ethnic minority status, and (iv) housing/transportation (**Table 1**). These variables are combined to produce both theme-specific and overall SVI measures, expressed as percentile rankings from 0.0 to 1.0. Higher SVI values indicating greater social vulnerability. Thus, an SVI score of 0.75 indicates that a community is more vulnerable than 75% of comparable geographic units. **Fig 1** illustrates the geographic distribution of overall SVI percentile rankings across North Carolina at the ZCTA level in 2022.

**Table 1:** CDC, social vulnerability index: Themes and variables.

No.	Major Theme	Variable(s)
1.	Socioeconomic Status	Below Poverty Level Unemployed Housing Cost Burden No High School Diploma No Health Insurance
2.	Household Characteristics	Aged 65 or Older Aged 17 or Younger Civilian with Disability Single Parent Household English Language Proficiency
3.	Racial & Ethnic Minority Status	Hispanic/Latino of any Race Black, Asian, American Indian, Alaska Native, Native Hawaiian, Pacific Islander, Two or more Races, all other Races*
4.	Housing Type & Transportation	% Multi-Unit Structures % Mobile Homes % Crowding (More people than rooms) % Households without a Vehicle % In Institutionalized Group Quarters

Hispanic/Latino ethnicity is reported separately and may include individuals of any race. All race categories shown excluding “Hispanic/Latino of any race” represent non-Hispanic/non-Latino individuals.

Source: CDC/ATSDR, 2022 [13].

SVI data, which included percentile rankings for all N.C. ZIP Code Tabulation Area (ZCTA) boundaries were obtained from the CDC website (https://www.atsdr.cdc.gov/place-health/php/svi/index.html). Residential ZIP Code of each decedent in the mortality data was linked to the corresponding ZCTA using the U.S. Department of Housing and Urban Development ZIP-to-ZCTA crosswalk file [17]. This process help to ensure spatial consistency for mapping and analytical comparability with census-derived population denominators when calculating mortality rates [18]. While small-area analyses may be susceptible to instability in sparsely populated rural ZCTAs, this approach was intentionally selected to capture within-county heterogeneity that would be obscured by coarser geographic units. To mitigate potential instability, we employed multiple complementary spatial analytic methods, including spatiotemporal cluster detection and both global and local measures of spatial autocorrelation, allowing for cross-validation of observed spatial patterns across independent techniques**.** Consistency of findings across methods was used as an indicator of robustness. Similar crosswalk and boundary harmonization approaches have been employed in global studies linking administrative and statistical boundaries to examine health inequities across diverse geographic settings.

The total number of COVID-19 deaths was aggregated at the ZCTA level and spatially represented using ZCTA geographic centroids for analysis in ArcGIS Pro (version 3.5.2; Esri, Redlands, CA). Unadjusted mortality rates (per 100,000 population) were calculated by dividing the number of deaths in each ZCTA (numerator) by the corresponding population (denominator) at the same scale, using data derived from the U.S. Census Bureau [19].

### Spatial analysis

#### Mortality waves.

Daily mortality counts were initially inspected using Microsoft Excel for preliminary exploration and quality checking, while final mortality wave plot was generated using Python 3.11.11 with the matplotlib library [20]. Guided by N.C. epidemiologic surveillance data, vaccine rollout timelines, and contemporaneous public health reports, the study period was divided into four different periods, or waves (**Table 2):** Pre-Vaccine (March–September 2020), Winter Surge (October 2020–February 2021), Delta (March–October 2021), and Omicron (November 2021–April 2022). This stratification allowed detection of heterogeneous temporal patterns in mortality similar to analytic approaches used globally to assess variant emergence, vaccine uptake, and policy interventions.

**Table 2:** Estimated start and end dates of significant COVID-19 pandemic waves, North Carolina (2020-2022).

Description	Estimated Wave Period
Pre-Vaccine (Initial) Wave	March 1, 2020, to September 30, 2020
Winter Wave	October 1, 2020, to February 28, 2021
Delta Wave	March 1, 2021, to October 31, 2021
Omicron Wave	November 1, 2021, to April 30, 2022

Source: NC, DHHS, 2020–2022.

#### Hot spot detection.

SaTScan™ (version 10.1), a spatial scan, cluster detection statistical software developed by the U.S. National Cancer Institute, was used to detect COVID-19 mortality clusters, or hotspot areas, both cumulatively over the entire study period and during each wave of the pandemic [21,22]. The purely spatial scan mode with a discrete Poisson probability model was used to compare observed deaths within each scanning window with those expected under spatial randomness. The maximum spatial cluster size was set to 50% of the population at risk, consistent with the conventional SaTScan^TM^ specification for purely spatial analyses to allow detection of both localized and broader regional mortality clusters across the state [21]. Because the maximum cluster size can affect cluster extent, sensitivity analyses using smaller upper bounds of population at risk (i.e., 10% and 25%**)** were also conducted; these produced substantively similar hotspot locations, with the expected reduction in cluster extent. Statistical significance was assessed using 999 Monte Carlo replications (p < 0.05**),** consistent with SaTScan^TM^ guidance recommending at least 999 replications to ensure excellent statistical power [20]. Cluster detection output from SatScan^TM^, was imported into ArcGIS Pro for mapping and visualization.

#### Spatial autocorrelation and bivariate analysis.

For all spatial autocorrelation analyses, including global and local Moran’s I, and bivariate Lee’s L statistics, spatial weights were constructed using a first-order contiguity (Queen’s case) approach, whereby ZCTAs were defined as neighbors if they shared a common boundary or vertex. This specification was selected to accommodate the irregular shape and size of ZCTAs and to reflect plausible neighborhood interactions without imposing an arbitrary distance threshold. The spatial weights matrix was row-standardized to ensure comparability across ZCTAs with differing numbers of neighbors and to stabilize variance in spatial dependence measures. No distance-based kernels or k-nearest neighbor (KNN) specifications were applied.

Spatial dependence of mortality and social vulnerability was assessed using Moran’s I in ArcGIS Pro (version 3.5.2; Esri, Redlands, CA). This analysis was selected to quantify whether each variable exhibited spatial clustering, dispersion, or random distribution across ZCTAs. For global Moran’s I, values range from –1, indicating complete spatial dispersion, to +1, indicating strong spatial clustering, and values near zero suggesting spatial randomness. Statistical significance was evaluated using 999 random permutations. While global Moran’s I assessed overall spatial structure, local Moran’s I was subsequently applied to identify localized patterns of variables by classifying ZCTAs as either High–High (hot spots), Low–Low (cold spots), or spatial outliers (High–Low and Low–High) [23].

To assess the strength and direction of spatial association between COVID-19 mortality and social vulnerability across ZCTAs, we applied Lee’s L statistic, a bivariate extension of Moran’s I [24]. Output values of Lee’s L statistic range from –1 to +1, with positive values indicating spatial co-location of high mortality and high social vulnerability, and negative values indicating inverse spatial relationships. Both global and local Lee’s L analyses were conducted using the Local Bivariate Relationship tool in ArcGIS Pro (version 3.5.2). Statistical significance was assessed using 999 random permutations, and a false discovery rate (FDR) correction (*p* < 0.05) was applied to local tests to account for multiple comparisons, with formal statistical significance defined as *p* < 0.05 [25]. ZCTAs were classified into four local spatial association categories: High–High (HH), High–Low (HL), Low–High (LH), or Low–Low (LL). This multi-scale approach enabled identification and visualization of localized areas where elevated COVID-19 mortality co-occurred with high social vulnerability. HH, HL, and LH clusters were interpreted using the primary FDR-adjusted conventional significance threshold of *p* < 0.05. LL clusters with *p* < 0.10 were retained and displayed separately as borderline, descriptive evidence of local low-risk spatial association and were not interpreted as meeting the primary threshold for statistical significance. This multi-scale approach enabled identification and visualization of localized areas where elevated COVID-19 mortality co-occurred with high social vulnerability, while also allowing descriptive assessment of weaker, low-risk spatial patterning. Similar methods have been widely applied in spatial epidemiologic studies examining inequitable distributions of disease risk [26–29].

#### Statistical analysis.

As a non-spatial complement to the spatial analytic methods (Moran’s, I and local bivariate Lee’s L), we conducted Mann–Whitney U tests to compare median SVI (percentile rankings) between ZCTA hot-spot areas and ZCTA non-hot spot areas. Effect sizes were expressed as rank-biserial correlation (r), interpreted as small (~0.1), medium (~0.3), or large (~0.5). Cohen’s *d* was estimated from *r* for additional interpretive context. Statistical significance was set at *p* < 0.05. Descriptive characteristics of COVID-19 decedents were summarized using frequencies and percentages. Differences in categorical distributions across study years were assessed using Pearson chi-square tests, a nonparametric method appropriate for comparing proportions across independent groups. All non-spatial statistical analyses were performed using SPSS (version 28.0). Mortality wave plot was generated using Python 3.11.11 with the matplotlib library [20]. Spatial analyses were conducted using the GIS and SaTScan^TM^ software platforms as described above.

## Results

Across the study period, a total of 25,051 COVID-19 deaths met our study criteria for analysis (**Table 3**). Notably, demographic and contextual differences were observed in sex distribution, shifting slightly over time, with males comprising a growing proportion of deaths, increasing from 51.8% in 2020 to 54.8% in 2021, and 53.6% in 2022 (p < .001). Racial patterns demonstrated significant changes: with White decedents consistently represented as the majority, noting an increase from 66.3% in 2020 to 74.2% in 2022. In contrast, the proportion of Black/African American decedents declined from 26.8% in 2020 to 19.6% in 2022. Smaller proportions were observed among American Indian/Alaska Native decedents, ranging from 1.3% to 1.6% across study years, and among individuals classified as Other or Unknown race, ranging from 4.1% to 5.6%, with both categories remaining relatively stable over time (p < .001). Age distribution tended to shift significantly (p < .001). In 2020, nearly half of COVID-19 deaths occurred among adults aged ≥80 years (49.5%), compared to 36.9% in 2021 and 40.5% in 2022. Meanwhile, the relative contribution of decedents aged 60–79 years increased across the study period, while deaths among those younger than 60 remained comparatively uncommon.

**Table 3:** Characteristics of COVID-19 decedents in North Carolina, 2020–2022 (N = 25,051).

		2020	2021	2022	p-value
Variable	Category	(n = 7,646)	(n = 13,138)	(n = 4,267)	
		n (%)	n (%)	n (%)	
Sex	Female	3685 (48.2)	5934 (45.2)	1981 (46.4)	<.001
	Male	3961 (51.8)	7204 (54.8)	2286 (53.6)	
Race	White	5064 (66.3)	9451 (71.9)	3167 (74.2)	<.001
	Black/Afr. Am.	2051 (26.8)	2934 (22.3)	837 (19.6)	
	AI/AN	100 (1.3)	215 (1.6)	65 (1.5)	
	Other/Unknown	431 (5.6)	538 (4.1)	198 (4.6)	
Age Group	<40 years	96 (1.3)	227 (1.7)	46 (1.1)	<.001
	40–49 years	161 (2.1)	395 (3.0)	122 (2.9)	
	50–59 years	426 (5.6)	1193 (9.1)	386 (9.1)	
	60–69 years	1,170 (15.3)	2627 (20.0)	846 (19.8)	
	70–79 years	2012 (26.3)	3846 (29.3)	1140 (26.7)	
	≥80 years	3781 (49.5)	4850 (36.9)	1727 (40.5)	
Marital Status	Married	2,786 (36.5)	5891 (44.8)	1810 (42.4)	<.001
	Widowed	2839 (37.1)	3637 (27.7)	1274 (29.8)	
	Div/Separated	1125 (14.7)	2163 (16.5)	748 (17.5)	
	Never Married	837 (11.0)	1381 (10.5)	411 (9.6)	
	Unknown	59 (0.8)	66 (0.5)	24 (0.6)	
Education*	<High School	1097 (14.4)	1287 (9.8)	421 (9.9)	<.001
	HS/GED	1124 (14.7)	1834 (14.0)	623 (14.6)	
	Some College	2946 (38.5)	5278 (40.2)	1766 (41.4)	
	Bachelor’s+	1433 (18.7)	3003 (22.9)	919 (21.5)	
	Unknown	1046 (13.7)	1736 (13.2)	538 (12.6)	
Place of Death	Inpatient	5074 (66.4)	9968 (75.9)	3175 (74.4)	<.001
	ER/Outpatient	159 (2.1)	318 (2.4)	112 (2.6)	
	Home	366 (4.8)	1,041 (7.9)	392 (9.2)	
	Hospice	264 (3.5)	466 (3.6)	218 (5.1)	
	Nursing/LTC	1686 (22.0)	1136 (8.6)	257 (6.0)	
	Other/Unknown	97 (1.3)	209 (1.6)	113 (2.6)	

*Includes, having some college = but no degree. Bachelors+ includes Masters, or PhD. Percentages reflect within-year distributions. P-values are from Pearson chi-square tests comparing category distributions across years. Exact values < .001 are reported as ‘<.001’. Years are calendar months and only includes days in the study period, (March 1, 2020, to April 30, 2022).

Source: NC, DHHS, 2020–2022.

Marital status patterns likewise changed significantly (*p* < .001). The proportion of married decedents increased from 36.5% in 2020 to 44.8% in 2021, with a slight decline in 2022 (42.4%). Conversely, widowed decedents declined from 37.1% in 2020 to 29.8% in 2022. Educational attainment also displayed statistically significant variation (p < .001). While having “some college” represented the highest percentage category across all years (38.5–41.4%), the proportion of decedents with less than a high school education declined from 14.4% in 2020 to 9.9% in 2022. Overwhelmingly, the reported, “place of death,” was “inpatient hospital deaths,” increasing over the study period from 66.4% in 2020 to 75.9% in 2021, with a slight decrease to 74.4% in 2022. Deaths occurring at home (4.8% to 9.2%) and in hospice (3.5% to 5.1%) rose across the period, while nursing home and long-term care facility deaths sharply declined, from 22.0% in 2020 to 6.0% in 2022.

### Mortality trends

**Fig 2:**
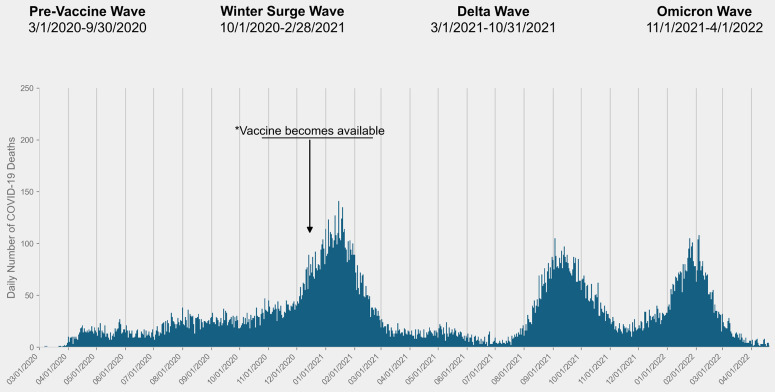
Number of COVID-19 deaths and estimated pandemic waves, North Carolina (March 2020 – April 2022). Notes: * COVID-19 vaccine roll out in N.C. (first administered to healthcare workers). Source: NCDHHS, SCHS, 2020-2022.

On average, the onset of the Pre-Vaccine Wave (March 1 – September 30, 2020) showed a relatively slight, yet stable upward increase of deaths, averaging approximately 19 deaths per day (**Fig 2).** In mid-July (2020), the number of daily deaths continued upward reaching a peak of approximately 38 deaths (per day) in the first weeks of August. This was followed by a downward dip of approximately 20 deaths per day towards the end of September. The Winter Surge Wave (October 1, 2020 – February 28, 2021), reported increased deaths in early October, and continued to rise before reaching a peak in the second week of January. This wave experienced the highest number of reported deaths (n = 141) across the entire pandemic period with approximately 141 deaths per day. Approximately one week following COVID-19 vaccine availability to healthcare workers (December 15, 2020), the number of deaths sharply declined to approximately 19 per day. After daily COVID-19 deaths peaked in early January 2021, deaths declined sharply and continued decreasing into the latter weeks of February (2021).

At the beginning of the third week of the Delta Wave (March 1 – October 31, 2021), the number of reported deaths remained relatively low. This trend continued and remained stable, averaging approximately 20 per day through the spring months (April, May, June). In early July, a sharp, yet brief decline of approximately five deaths per day occurred before climbing early to mid-August, reaching the second highest peak of the pandemic of approximately 105 deaths per day. In late September, a downward trend continued and stabilized to around 18 deaths per day through late October (2021).

At the onset of the Omicron Wave (November 1, 2021 – April 30, 2022), the number of daily deaths increased, reaching its overall third peak in January-February at levels comparable to the Delta wave (~100 per day). However, unlike the Delta wave, the Omicron surge was shorter in duration, with mortality declining sharply by early February and stabilizing at fewer than 10 deaths per day by early April. This trend marked a notable transition into the post-Omicron period.

### Hot spot analysis

**Fig 3:**
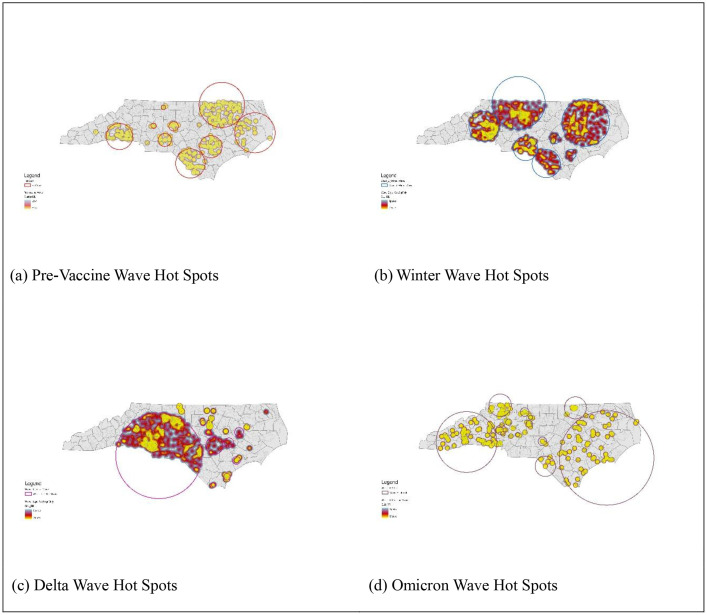
Heat map of COVID-19 mortality hot spots detected at four different waves of the pandemic, North Carolina (2020-2022). Note: This figure was created by the authors using ArcGIS Pro and is based exclusively on publicly available U.S. federal and state spatial data; no proprietary basemap or copyrighted imagery was used.

As shown in **Fig 3**, significant spatial clustering of COVID-19 mortality was observed across all four pandemic waves, though the number, location, and intensity of hot spots varied considerably over time. During the Pre-Vaccine Wave, mortality clusters were identified across the state with a statistically significant of deaths concentrated along the south-central border (known as the Sandhills region), the Charlotte metropolitan region, and the upper mid-northeast region (RR = 1.84–4.83; *p* < .001) reflecting geographically concentrated but relatively homogeneous excess mortality. In the Winter Surge, the number of deaths rose significantly, and became more concentrated in existing hot spot areas while the northern central border region emerged (S1 Table). Clustering strength peaked during the Winter wave, but relative risks of hot spots areas were narrower than the initial wave (RR = 1.21–2.39; *p* < .001), indicating deaths were increasingly more uniform across the state. By contrast, the Delta Wave witnessed highly concentrated mortality clustering (RR = 1.94–84.36; *p* < .001) in the southwestern regional border of the state (i.e., Mecklenburg County and surrounding area). In the final Omicron Wave, mortality hot spots were dispersed across the state, with the most significant clustering (RR = 1.82–13.54; *p* < .001) seen largely in the far eastern region (i.e., Outer and Inner Banks) and far western counties, indicating that while mortality was elevated statewide, distinct high-risk areas persisted in regions of structural vulnerability and limited healthcare access.

### Spatial autocorrelation

#### Moran’s I.

Overall, Global Moran’s I test identified significant spatial clustering of mortality and SVI within high-mortality ZCTAs, but not statewide **(**S1 Fig and S2 Table**)**. All SVI measures demonstrated significant, though more modest, spatial autocorrelation, with the SVI minority status/language domain showing the strongest clustering (Moran’s I = 0.082, z = 4.70*, p* < 0.001), followed by socioeconomic status, household composition/disability, and housing/transportation. Although statewide mortality hot spots demonstrated spatial dependence (Moran’s I = 0.073, z = 6.53, *p* < 0.001), socially vulnerable areas were largely spatially random (all *p* > 0.50).

#### Lee’s L statistics.

**Fig 4:**
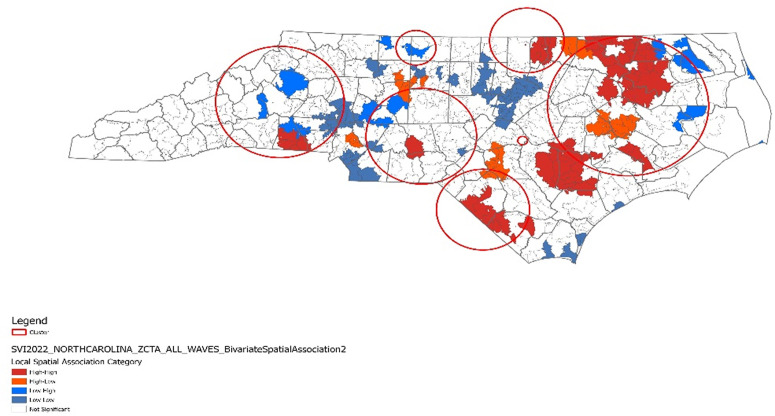
Bivariate analysis of COVID-19 mortality and socially vulnerable areas in North Carolina (2020–2022). Notes: Bivariate Lee’s L analysis illustrating statistically significant local spatial associations between COVID-19 mortality rates and Social Vulnerability Index (SVI) percentile ranking at the ZIP Code Tabulation Area (ZCTA) level. Mortality hot spots detected across the full study period (March 1, 2020–April 30, 2022). HH, HL, and LH clusters met the primary significance threshold of *p* < 0.05; LL clusters are shown at *p* < 0.10 as borderline/descriptive local spatial associations. This figure was created by the authors using ArcGIS Pro and is based exclusively on publicly available U.S. federal and state spatial data; no proprietary basemap or copyrighted imagery was used.

In global bivariate analysis, the spatial relationship between COVID-19 mortality and social vulnerability demonstrated a positive and statistically significant spatial association, indicating that ZCTAs with higher social vulnerability tended to be geographically co-located with neighboring areas experiencing higher COVID-19 mortality. This association was quantified using the global bivariate Lee’s L statistic, which measures spatial co-location rather than explained variance. In more refined local analyses (**Table 4).** Local bivariate Lee’s L statistics identified significant high–high (HH) (p < 0.05) clustering, reflecting areas where elevated COVID-19 mortality co-occurred with high social vulnerability (**Fig 4).**

**Table 4:** Local Bbivariate Lee’s *L* cluster categories, significance thresholds and interpretation, North Carolina, 2020–2022.

Cluster Category	Spatial Relationship	Lee’s L	*p*-value	Interpretation	*ZCTAs (n)
High–High (HH)	High COVID-19 mortality surrounded by high social vulnerability	Positive	*p* < 0.05	Strong positive local association; significant hot spots of co-occurrence	34
High–Low (HL)	High COVID-19 mortality surrounded by low social vulnerability	Negative	*p* < 0.05	Inverse association; high-mortality outliers in otherwise low-vulnerability neighborhoods	23
Low–High (LH)	Low COVID-19 mortality surrounded by high social vulnerability	Negative	*p* < 0.05	Inverse association; resilient or low-mortality areas within high-vulnerability surroundings	8
Low–Low (LL)	Low COVID-19 mortality surrounded by low social vulnerability	Positive	*p* < 0.10	Borderline/descriptive positive low association; low-risk clusters	54
Not Significant	No statistically meaningful spatial relationship	—	*p* ≥ 0.10	Random spatial pattern	368

Values represent the number of ZCTAs (n) classified into each local bivariate spatial association category based on Lee’s L statistics. Local spatial analyses were restricted to ZCTAs with complete COVID-19 mortality and SVI data and sufficient spatial contiguity. HH, HL, and LH categories were interpreted using the primary significance threshold of *p* < 0.05. LL clusters are displayed at *p* < 0.10 and are interpreted as borderline/descriptive patterns of low-risk spatial association rather than as findings meeting the primary threshold for statistical significance.

Low–low clusters (LL) were also observed and are presented as borderline/descriptive patterns (*p* < 0.10) of lower-risk spatial association; these did not meet the primary significance threshold applied to the other local cluster categories. Conversely, high–low (HL) and low–high (LH) outliers (*p* < 0.05) indicated localized spatial mismatches between mortality and vulnerability.

In nonparametric analysis, social vulnerability, particularly racial/ethnic composition and socioeconomic disadvantage was consistently higher in mortality hot spots than in non-hot spot areas (**Table 5).** However, the moderate magnitude and spatial extent of bivariate Lee’s L values indicate only a modest spatial association, suggesting that social vulnerability is spatially correlated with only part of the observed spatial variation in COVID-19 mortality.

**Table 5:** Non-parametric analysis of social vulnerability and COVID-19 hot spots vs. non-hot spots, North Carolina (2020-2022).

Social Vulnerability Domain	Group	n	Median [IQR]	U	Z	p-value	r
Socioeconomic Status	Cluster	342	0.61 [.34–.82]	69363.5	−4.656	<.001	0.16
	Non-cluster	500	0.47 [.23–.72]				
Household Characteristics	Cluster	342	0.57 [.31–.80]	72400.0	−3.780	<.001	0.13
	Non-cluster	500	0.48 [.22–.73]				
Race & Ethnicity	Cluster	342	0.54 [.32–.81]	69029.5	−4.753	<.001	0.16
	Non-cluster	500	0.39 [.17–.70]				
Housing & Transportation	Cluster	342	0.56 [.31–.79]	74286.5	−3.236	<.001	0.11
	Non-cluster	500	0.46 [.19–.75]				
Composite SVI Score	Cluster	342	0.58 [.39–.74]	66718.5	−5.419	<.001	0.19
	Non-cluster	500	0.45 [.28–.68]				

Abbreviations: IQR = interquartile range; U = Mann–Whitney U statistic; Z = standardized test statistic; r = effect size. Composite SVI Score reflects an overall composite index value provided by CDC/ATSDR.

## Discussion

This study demonstrated that COVID-19 mortality in North Carolina was not randomly distributed. Across the pandemic period, mortality hot spots were consistently concentrated in rural, socioeconomically disadvantaged, and healthcare-limited areas. Using multiple complementary spatiotemporal analytic methods, we identified substantial geographic heterogeneity in mortality patterns, with a disproportionate burden borne by socially vulnerable communities. To our knowledge, this analysis represents one of the most comprehensive spatial epidemiological assessments of COVID-19 mortality conducted in the state. These findings align with prior research documenting spatial inequities in COVID-19 morbidity and mortality associated with poverty, crowded housing, minority status, limited English proficiency, disability, and constrained access to healthcare infrastructure [3,6,26–28]. Collectively, this evidence underscores that the spatial concentration of COVID-19 deaths reflects enduring structural determinants of respiratory health rather than isolated or context-specific phenomena.

Nonparametric test results showed statistically significant but modest differences in social vulnerability between mortality hot spots and non–hot spot areas. These findings indicate that social vulnerability is associated with only part of the observed spatial heterogeneity in COVID-19 mortality. The modest effect sizes suggest that additional contextual factors including healthcare access, comorbidity burden, occupational exposure, long-term care facility density, and variation in vaccination uptake, also shaped localized mortality patterns, particularly in rural settings [27–32]. Wave-specific analyses further revealed both spatial and temporal variation in mortality distribution over the two-year study period. Earlier pandemic waves disproportionately affected rural and socioeconomically disadvantaged areas, consistent with limited healthcare access and baseline vulnerability. In contrast, the Delta wave exhibited greater geographic heterogeneity, while the Omicron wave, though less severe overall maintained distinct and stable spatial clustering. These findings parallel U.S. and international studies demonstrating that pandemic risk shifted over time in response to viral evolution, behavioral adaptations, vaccination uptake, and public health interventions [29,30]. Importantly, the persistence of spatial clustering across waves suggests that structural vulnerability continued to shape outcomes even as the epidemiologic context evolved.

Taken together, the observed spatial patterns extend beyond descriptive mapping to highlight meaningful intersections between mortality clustering and social vulnerability. Persistent hot spots, observed in North Carolina and elsewhere, point to the need for sustained investments in healthcare infrastructure, chronic disease management, and broadband capacity to support telehealth and remote care [26,31]. Late-emerging clusters further underscore the importance of continuous surveillance and adaptive health system capacity. The consistent overlap between mortality clustering and social vulnerability reinforces the necessity of equity-driven, geographically informed strategies. These approaches are increasingly operationalized through global initiatives such as the World Health Organization’s Health Inequality Data Repository and pandemic early warning systems, which emphasize the integration of health outcomes with structural and social determinants [32].

Overall, the pandemic revealed critical lessons for public health practice and policy. Gaps in data integration and surveillance infrastructure underscored the need for timely, granular systems capable of linking mortality, vaccination, and comorbidity data. Persistent disparities further highlighted the importance of culturally competent communication and trust-building to improve vaccine uptake and access to care [27,28]. Globally, hospitals and clinicians learned that effective surge capacity planning must incorporate spatial intelligence to anticipate intensive care demand and workforce needs [29]. Together, these lessons emphasize that integrating fine-scale spatial analysis with equity-informed public health systems is essential for strengthening preparedness, guiding resource allocation, and informing future analytic extensions including spatial regression and non-stationarity assessments in larger-scale studies [29].

Within this context, our findings underscore the value of regionally responsive public health policies supported by early-warning systems that inform decision-making rather than merely describe risk [30–33]. For example, space–time analytic tools offer a practical way for identifying when and where resources should be mobilized as conditions evolve. Focusing on areas characterized by persistent high mortality and elevated social vulnerability can also serve as surveillance triggers for targeted, actionable interventions, including surge vaccination, telehealth expansion, transportation support, temporary workforce augmentation, and deployment of mobile healthcare units [34]. Compared with uniform statewide approaches, regional strategies enable faster, more precise action where health system capacity is most constrained, supporting timely and equitable resource allocation. Collectively, these findings reinforce the importance of embedding spatial intelligence into routine policy frameworks and prioritizing long-term structural investments such as broadband access, community health workers, and disease prevention to strengthen preparedness for future pandemics. Future research could extend this work using spatial regression and spatially varying coefficient models, such as geographically weighted regression, to quantify explained variance, assess robustness after covariate adjustment, and formally evaluate spatial non-stationarity particularly in larger-area or multi-state analyses where covariate stability and model assumptions can be more fully evaluate [34,35].

### Limitations

Several limitations should be noted. First, while ZIP Codes and ZIP Code Tabulation Areas (ZCTAs) are often used interchangeably, they are not perfectly aligned; a single ZIP Code may map to multiple ZCTAs, and nonresidential or Post Office Box codes were excluded to preserve analytic accuracy. The use of ZCTA-level geography represents a deliberate tradeoff between spatial resolution and statistical stability, particularly in sparsely populated rural areas where small population denominators may introduce variability. Nevertheless, ZCTAs were selected because they aligned with the reporting structure of both the mortality and SVI datasets and enabled detection of within-county heterogeneity that would otherwise be masked in county-level analyses. An additional strength of SaTScan’s discrete Poisson model, is that it accounts for the underlying population at risk, thereby reducing the likelihood that clusters were driven solely by uneven population size. However, this approach does not fully resolve instability in sparsely populated small areas. To address this concern, we used multiple complementary spatial analytic methods, and observed consistent, convergent spatial patterns across approaches. Together, these findings support the overall robustness of the identified spatial patterns, although some instability may remain in low-population areas. Future studies should incorporate population thresholds, empirical Bayes or other rate-smoothing approaches, and formal sensitivity analyses to further evaluate the robustness of spatial clusters under alternative small-area assumptions [35,36].

Second, age-standardized ZCTA-level rates were not calculated because COVID-19 deaths in our data were overwhelmingly concentrated among adults aged ≥60 years. Further, age stratification at the ZCTA level would likely have introduced additional instability due to sparse denominators in small areas. Accordingly, age-adjusted spatial modeling represents an important consideration and important direction for future research.

Third, mortality was assigned to the decedent’s residential ZIP Code rather than place of death, which may obscure patterns related to hospital or referral center clustering. Fourth, as an ecological study, causal inference is not possible; observed associations at the ZCTA level should not be assumed to represent individual-level effects. Finally, although the SVI is robust and widely adopted, it was designed primarily for natural disaster contexts and may not fully capture pandemic-specific risk factors such as occupational exposure, health literacy, informal labor, political ideology, vaccine hesitancy, or underlying comorbidities [27,37–39].

## Conclusions

COVID-19 mortality in North Carolina exhibited distinct and shifting spatial clustering aligned with social vulnerability characteristics. Viewed within a broader context, these findings reinforce that place matters and that geographic and social inequities are universal drivers of disease burden. Applying spatial analysis frameworks that integrate epidemiology and social vulnerability metrics can strengthen pandemic preparedness and response at state, national, and global levels. Future research should extend this work through comparative cross-country analyses and longitudinal models integrating real-time surveillance, vaccination coverage, and healthcare utilization data [33,34]. Expanding vulnerability indices to include globally relevant dimensions, such as occupational risk, migration status, and access to preventive care, may further improve predictive capacity and support more equitable, targeted interventions [35–39].

## Supporting information

S1 Fig COVID-19 mortality hot spots (includes all pandemic waves over the study period; (March 2020-April 2022). (DOCX)

S1 Table COVID-19 mortality hot spots, North Carolina, 2020–2022. (DOCX)

S2 Table Univariate tests of global Moran’s I spatial autocorrelation for COVID-19 mortality rates and social vulnerability index rankings (SVI), North Carolina (2020–2022). (DOCX)

## Abbreviations

CDC: Centers for Disease Control and Prevention
SVI: Social Vulnerability Index.
