# Supplementary material for: Social vulnerability and spatial patterns of COVID-19 mortality: Global implications for respiratory health equity
Source: PLoS One. 2026 Jul 1;21(7):e0352270. doi: 10.1371/journal.pone.0352270 (PMC13322539; doi:10.1371/journal.pone.0352270)
Supplement: S1 Fig — (DOCX) [file pone.0352270.s001.docx]

**
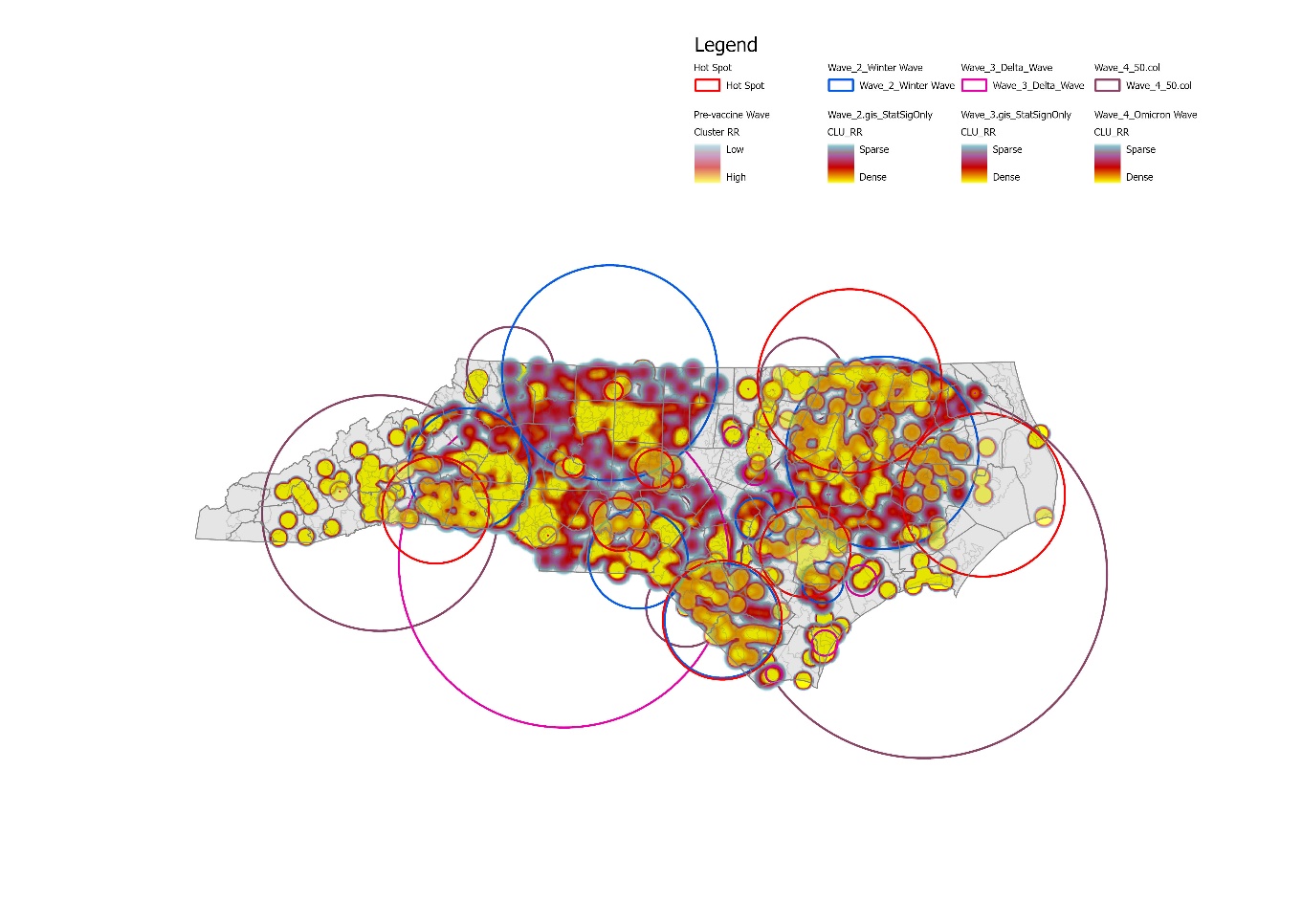
**

**Figure S1.** COVID-19 Mortality Hot Spots (includes all pandemic waves over the study period; March 2020-April 2022)

Note: This figure was created by the authors using ArcGIS Pro and is based exclusively on publicly available U.S. federal and state spatial data; no proprietary basemap or copyrighted imagery was used.
