# Supplementary material for: Social vulnerability and spatial patterns of COVID-19 mortality: Global implications for respiratory health equity
Source: PLoS One. 2026 Jul 1;21(7):e0352270. doi: 10.1371/journal.pone.0352270 (PMC13322539; doi:10.1371/journal.pone.0352270)
Supplement: S1 Table — (DOCX) [file pone.0352270.s002.docx]

| **Table S1**. COVID-19 Mortality Hot Spots, North Carolina, 2020-2022 | | | | |
| --- | --- | --- | --- | --- |
| Wave | Hot Spots  (n) | Regions  Affected | p-value | RR  (range) [IQR] |
| Pre-Vaccine | 12 | South central Sandhills, Charlotte metro, mid-northeast | < .001 | 1.84–4.83 [1.04] |
| Winter Surge | 7 | Northeastern, northern border, south-central | < .001 | 1.21–2.39 [0.33] |
| Delta | 20 | Southwestern/Mecklenburg area, extended regions | < .001 | 1.94–84.36 [9.53] |
| Omicron | 16 | (Western Counties-Madison–Cherokee, Clay, Graham, Swain ) | < .001 | 1.82–13.54 [4.96] |
