# Supplementary material for: Social vulnerability and spatial patterns of COVID-19 mortality: Global implications for respiratory health equity
Source: PLoS One. 2026 Jul 1;21(7):e0352270. doi: 10.1371/journal.pone.0352270 (PMC13322539; doi:10.1371/journal.pone.0352270)
Supplement: S2 Table — (DOCX) [file pone.0352270.s003.docx]

| **Table S2.** Univariate Tests of Global Moran’s I Spatial Autocorrelation for COVID-19 Mortality Rates and Social Vulnerability Index Rankings, North Carolina (2020–2022) | | | | | | | | | | | | | | | | | |  |
| --- | --- | --- | --- | --- | --- | --- | --- | --- | --- | --- | --- | --- | --- | --- | --- | --- | --- | --- |
| Major SVI Theme | Mortality Rates  (Within hot spots) | | | | | | | |  | | Mortality Rates  (State-wide) | | | | | | |  |
|  | Moran’s I | | | *z* | *p* | | Pattern | |  | | Moran’s I | | | z | | *p* | Pattern | |
| Socioeconomic status | | 0.07 | 3.9 | | | <.001 | | Clustered | |  | | –0.02 | –0.7 | | .512 | | Random | |
| Household characteristics | | 0.06 | 3.7 | | | <.001 | | Clustered | |  | | –0.01 | –0.3 | | .735 | | Random | |
| Racel & ethnicity | | 0.08 | 4.7 | | | <.001 | | Clustered | |  | | –0.01 | –0.2 | | .849 | | Random | |
| Housing /transportation | | 0.06 | 3.4 | | | <.001 | | Clustered | |  | | –0.03 | –0.3 | | .735 | | Random | |
| SVI (all themes) | | 0.07 | 3.9 | | | <.001 | | Clustered | |  | | –0.03 | –0.7 | | .512 | | Random | |
| *Mortality rates were calculated for each ZCTA-Code | | | | | | | | | | | | | | | | | |  |
